# Provision of postpartum care to women giving birth in health facilities in sub-Saharan Africa: A cross-sectional study using Demographic and Health Survey data from 33 countries

**DOI:** 10.1371/journal.pmed.1002943

**Published:** 2019-10-23

**Authors:** Lenka Benova, Onikepe Owolabi, Emma Radovich, Kerry L. M. Wong, David Macleod, Etienne V. Langlois, Oona M. R. Campbell

**Affiliations:** 1 Faculty of Epidemiology and Population Health, London School of Hygiene and Tropical Medicine, London, United Kingdom; 2 Department of Public Health, Institute of Tropical Medicine, Antwerpen, Belgium; 3 Guttmacher Institute, New York City, New York, United States of America; 4 Alliance for Health Policy and Systems Research, Science Division, World Health Organization, Geneva, Switzerland; University of Manchester, UNITED KINGDOM

## Abstract

**Background:**

Postpartum care has the potential to avert a substantial proportion of maternal and perinatal mortality and morbidity. There is a crucial gap in understanding the quality of postpartum care for women giving birth in health facilities in low- and middle-income settings. This is particularly the case in sub-Saharan Africa (SSA), where the levels of maternal and neonatal mortality are highest globally despite rapid increases in facility-based childbirth. This study estimated the percentage of women receiving a postpartum health check following childbirth in a health facility in SSA and examined the determinants of receiving such check.

**Methods and findings:**

We used the most recent Demographic and Health Survey (DHS) conducted in 33 SSA countries between 2000–2016. We estimated the percentage of women receiving a postpartum check by a health professional while in the childbirth facility and the associated 95% confidence interval (CI) for each country. We analyzed determinants of receiving such checks using logistic regression of the pooled data. The analysis sample included 137,218 women whose most recent live birth in the 5- year period before the survey took place in a health facility. Of this pooled sample, 65.7% of women were under 30 years of age, 85.9% were currently married, and 57% resided in rural areas. Across countries, the median percentage of women who reported receiving a check was 71.7%, ranging from 26.6% in Eswatini (Swaziland) to 94.4% in Burkina Faso. The most fully adjusted model showed that factors from all four conceptual categories (obstetric/neonatal risk factors, care environment, and women’s sociodemographic and child-related characteristics) were significant determinants of receiving a check. Women with a cesarean section had a significantly higher adjusted odds ratio (aOR) of 1.88 (95% CI 1.72–2.05, *p* < 0.001) of receiving a check. Women giving birth in lower-level public facilities had lower odds of receiving a check (aOR 0.94, 95% CI 0.90–0.98, *p* = 0.002) compared to those in public hospitals, as did women attended by a nurse/midwife (compared to doctor/nonphysician clinician) (aOR 0.74, 95% CI 0.69–0.78, *p* < 0.001). This study was limited by the accuracy of the respondent’s recall of the provider, timing, and receipt of postpartum checks. The outcome of interest was measured using three slightly different question sets across the 33 included countries.

**Conclusions:**

The suboptimal levels of postpartum checks in health facilities in many of the included SSA countries partially reflect the lack of importance given to postpartum care in the global discourse on essential interventions and quality improvement in maternal health. Addressing disparities in access to both facility-based childbirth and good-quality postpartum care in SSA is critical to addressing stalling declines in maternal mortality and morbidity.

## Abstract 2

Lenka Benova and colleagues assess postpartum care in sub-Saharan Africa.

## Author summary

**Why was this study done?:**

- Between 20% and 44% of maternal deaths in low-resource settings are estimated to occur in the postpartum period.
- Understanding the care women receive after giving birth in health facilities is increasingly important given that more than half of births in sub-Saharan Africa now occur in health facilities.
- This study was conducted because there is a gap in the evidence on the provision of care to women delivering in health facilities in low- and middle-income countries.

**What did the researchers do and find?:**

- We analyzed data from 137,218 women who recently delivered a live baby in a health facility in 33 sub-Saharan African countries and assessed the percentage of women who reported that a health professional checked on their health before they were discharged.
- We found that the percentage of women receiving checks before discharge varied widely across all countries (from 27% in Eswatini to 94% in Burkina Faso) and was far from universal in most countries.
- Women who had a cesarean section were more likely to have been checked, while women who delivered at lower-level public facilities (versus a public hospital) or with a nurse/midwife (versus a doctor) were less likely to have received a check.

**What do these findings mean?:**

- Our results highlight the need for a greater attention to the provision of high-quality postpartum care in facilities across sub-Saharan Africa if the currently high levels of maternal mortality are to be tackled effectively.
- One challenge of our study was the subjective definition of a health check and the three different ways women were asked to report this across all countries. The checks and the person who provided them were self-reported by women and are hence dependent on how accurately they understood and remembered the circumstances after their births.
- The main implication of our study is that the lack of postpartum checks, combined with the short time women tend to stay in health facilities after birth in sub-Saharan Africa, likely contributes to underdiagnosis and poor management of some birth complications such as postcesarean surgical site infection.

## Introduction

An estimated 303,000 women died during pregnancy or following childbirth in 2015 [1]. In addition, 2.5 million neonatal deaths occur yearly, accounting for almost half (47%) of global deaths under 5 years of age [2]. Sub-Saharan Africa (SSA) is the region with the highest maternal mortality ratio and one of the highest neonatal mortality rates [3]. Most maternal and neonatal deaths occur at the time of childbirth or shortly after and decline exponentially with increasing time postpartum. The vast majority of maternal deaths, including those occurring postpartum, are treatable and preventable with timely recognition and good-quality care [4]. Despite the fundamental role played by postnatal care to ensure mothers and babies survive and thrive, postnatal services have the lowest median national coverage of interventions on the continuum of maternal and child healthcare [5].

Increasing the proportions of women in low- and middle-income countries (LMICs) giving birth in facilities could potentially ameliorate this situation by ensuring good-quality care during birth and the immediate postpartum period [6,7]. For babies, postnatal checks aim to ensure the baby is feeding well and involve measuring vital signs to ensure they are normal, examining the umbilical cord stump, and examining for sepsis and jaundice. For women, checks typically involve measuring vital signs, asking if the woman is experiencing physical symptoms that may indicate severe conditions, checking if the uterus is contracting well, examining vaginal tears/discharge or cesarean incisions, assessing ability to urinate and defecate, and conducting other tests based on the woman’s medical history [8].

The World Health Organization (WHO) recommends that women undergoing uncomplicated vaginal births in health facilities remain there for at least 24 hours after birth [8,9]. Checks provided during this critical period should enable health providers to detect the presence of conditions with a very high risk of maternal mortality and morbidity such as postpartum hemorrhage, puerperal infection, postpartum preeclampsia, and thromboembolism and of neonatal mortality such as sepsis before women and newborns are discharged. Women can also be informed of danger signs for which to seek treatment during the first few weeks after birth. A minimum 24-hour stay also opens an avenue for healthcare professionals to provide counseling on nutrition, breastfeeding, and postpartum contraception use [8,10].

Most of the current literature on coverage and quality of postpartum care focuses on women who gave birth outside of health facilities and on their newborn [11–13]. Much less is known about the quality of care for women within health facilities during the immediate postpartum period before discharge. Some studies even assume that all women giving birth in health facilities receive postpartum care before discharge [14]. However, the discrepancy between increasing levels of facility births but noncommensurate declines in maternal and neonatal mortality have highlighted that we cannot assume good quality of intrapartum and postpartum care in health facilities [15–17]. A recent study showed that the postpartum length of stay (LOS) in facilities might be too short in many countries to identify and treat complications [18].

In light of the gap in evidence on the coverage and quality of postpartum care for mothers giving birth in health facilities in LMICs, the objective of this study is to examine the proportion of women receiving a postpartum health check by a health professional before discharge from a health facility following childbirth in SSA using the most recent Demographic and Health Survey (DHS) data and to understand the determinants of receiving such postpartum checks.

## Methods

### Data

DHSs are cross-sectional, nationally representative household surveys, usually covering 5,000 to 30,000 households, and respondents are women of reproductive age (15–49 years). We used the most recent DHS data set as of December 2016 for each country in SSA that conducted a DHS survey since 2000 and collected information on the outcome of interest. The DHSs use a multilevel cluster sampling survey design; individual women’s survey weights are needed in analysis to adjust for this and for nonresponse.

### Population

All women aged 15–49 with a live birth in the survey recall period (5 years) were included in the analysis. We examined women’s self-reported postpartum care for the most recent birth in the recall period if the birth occurred in a health facility. In Kenya, women in a random half of sampled households were administered a short questionnaire that excluded questions on postpartum care; we included only the subsample of women in Kenya answering the full questionnaire. We excluded Mozambique because while questions were asked on the survey, the relevant variables were not in the data set.

### Definitions

Our primary outcome was a binary variable capturing whether a woman reported receiving a postpartum check from a health professional while in the childbirth facility. The construction and name of this variable reflects the wording of the series of questions asked to women on DHSs, with a conceptual link to WHO postnatal care guidelines that state that all women giving birth in health facilities should receive a pre-discharge check [8]. DHS have used several versions of questions to ask about such checks. We constructed the outcome variable from the three patterns of questions asked as shown in Table 1 (a full list of countries, survey years, and question patterns is available in S1 Table). We used country-level DHS categorizations of cadres considered to be “health professionals.” Less than 1% of women were missing responses for receipt of the check or cadre of provider; such cases were recoded as not having had a postpartum check from a health professional while in the childbirth facility.

**Table 1:** Sets of questions and responses used to define whether a woman received a postpartum check while in the facility following childbirth.

Pattern (Number of Countries)	Fulfilled Criteria for Having Outcome If
A (24)	Did anyone check on your health while you were still in the facility? = yes
	AND
	Who checked on your health at that time? = health professional
B (3)	Before you were discharged after (NAME) was born, did a health professional conduct a physical examination on you? = yes
	AND
	Who checked on your health at that time? = health professional
C (6)	Did anyone check on your health after you gave birth to (NAME)? = yes
	AND
	Who checked on your health at that time? = health professional
	AND
	Timing of the first check after delivery was before/on the day of discharge from facility (How long after delivery did the first check take place? ≤ LOS in facility) (calculated in days, not hours)

**Abbreviations**: LOS, length of stay.

We converted LOS at the health facility and timing of discharge check to hours based on the methods described in Campbell and colleagues [18]. For pattern C, we subtracted LOS from the timing of the postpartum check to determine when the check took place relative to discharge from the health facility. If the check occurred on the same day postpartum or earlier, we considered women to have received the check before discharge. If the check was later or if the timing variable was missing or recorded as “don’t know,” women were considered to have received their postpartum check after discharge from the facility.

**Fig 1:**
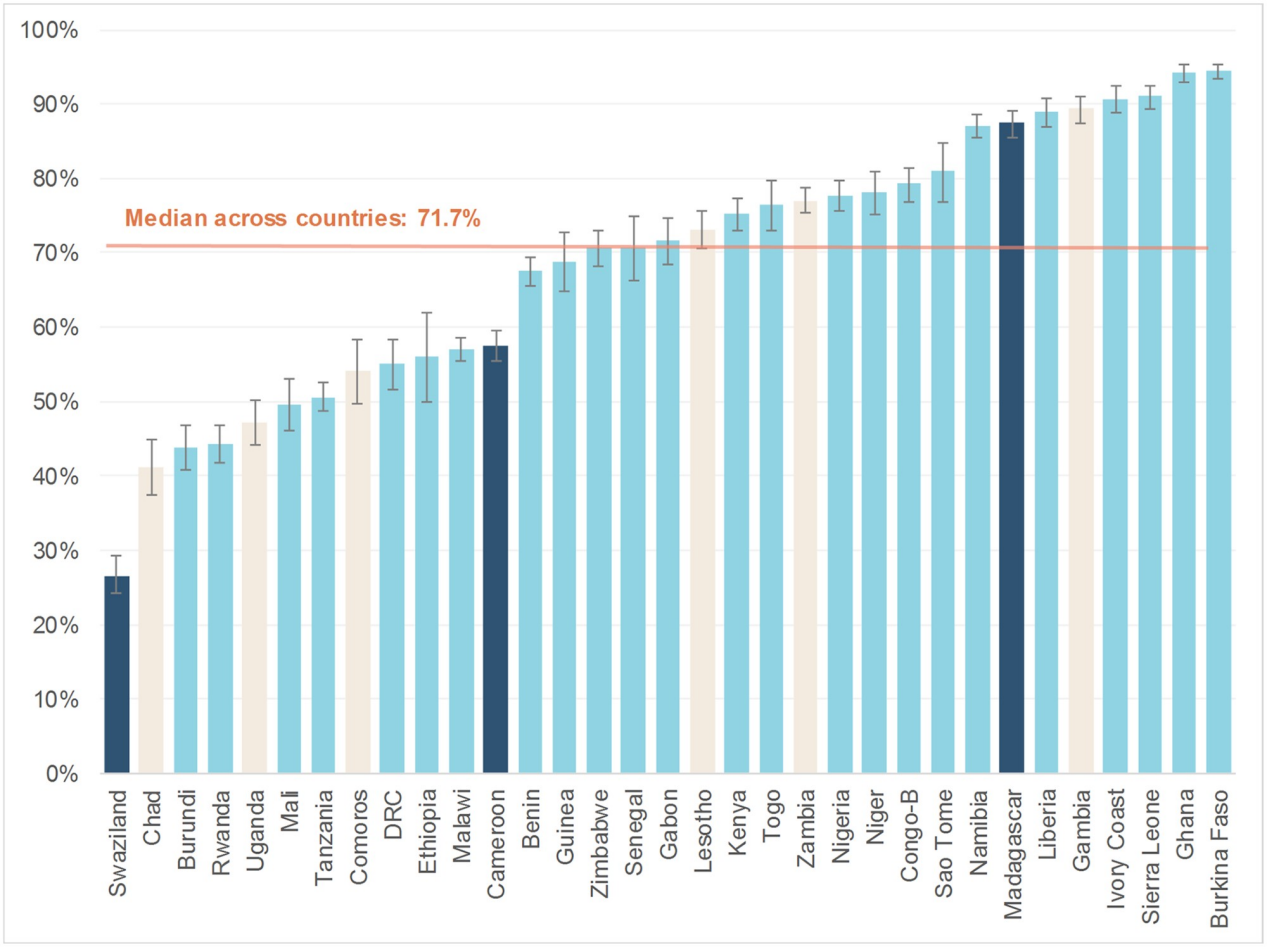
Conceptual categories of factors associated with receiving a postpartum check while in the facility following childbirth.

We grouped determinants of receiving a pre-discharge check into four categories of factors (Fig 1): obstetric and neonatal risk factors, women’s sociodemographic characteristics, child-related characteristics, and characteristics of the care environment. Some determinants may fall into multiple conceptual categories constructed to assess how risk factors might act on the outcome. For the purpose of this analysis, we identified three main determinants (place of residence, number of antenatal care [ANC] visits during pregnancy, and child’s birthweight) that fit in more than one conceptual category.

We assessed four obstetric/neonatal risk factors. Mode of delivery (vaginal or caesarean) and multiple birth (singleton or not) were categorized into binary variables. Next, we considered parity (first birth, 2–3, 4–6, and ≥7) and neonatal survival (survived, died before/on day of discharge, or died after discharge based on Campbell and colleagues [18]) as categorical variables. Child’s birthweight and number of ANC visits during pregnancy might reflect both obstetric/neonatal risk factors as well as the characteristics of the care environment. We considered whether the child was weighed as a reflection of the care environment (baby weighed at birth or not) in the main analysis. In sensitivity analyses expanding the conceptual link to obstetric/neonatal risk factors, we further disaggregated the child’s birthweight into categories based on WHO and consensus definitions of very low (<1,499 g), low (1,500–2,499 g), normal birthweight (2,500–4,000 g), and macrosomia (>4,000 g) [19]. We assessed ANC utilization during the index pregnancy by including a continuous variable capturing the number of visits reported by women (0, 1, 2, …, 10+).

For the care environment, we classified childbirth facilities into three categories: public hospital, public lower-level facilities, and nonpublic facility (all levels) because the levels of health facilities (lower-level versus hospitals) in the nonpublic sector were conflated in DHS response options in most countries. Nonpublic facilities included various types of providers outside of the public sector, including for-profit, nongovernmental organizations, and faith-based. Categories of highest level of attendant at birth were created to differentiate doctor/nonphysician clinician, other skilled birth attendant (SBA, comprising nurses, midwives, and auxiliary midwifery staff), and non-SBA based on provider and attendant groupings used in Benova and colleagues (2015) [20]. The care and support environment during childbirth and the immediate postpartum period was also captured through optimal breastfeeding practices; we defined immediate breastfeeding initiation as occurring within 1 hour of birth. Eight countries did not ask women for the length of time they stayed at the facility after childbirth. Within countries that did, <1% of respondents with available LOS data reported staying >21 days; these observations were recoded as equal to 21 days.

For women’s sociodemographic characteristics, we assessed mother’s age at birth, highest completed level of education, marital status, and household wealth quintiles derived by the DHS from household assets. Conceptually, place of residence (urban, rural) was also considered a reflection on the care environment. Child-related factors included sex (male, female) and mother’s desire for another child at the time of pregnancy/wantedness of the child (wanted at the time, wanted later = mistimed, not want another child = unwanted).

### Analysis

Analyses were conducted in Stata/SE v15. We present the percentages of women receiving a postpartum check while in the childbirth facility (and the associated 95% confidence intervals [CIs]) for each country adjusted for survey-specific weighting, clustering, and stratification. We pooled the data from all countries to show descriptive statistics and weighted observations by a combination of country-specific survey weights and the country’s population based on United Nations population estimates for the median survey year of 2012.

We used logistic regression of the pooled data (without survey setting adjusting for weighting, clustering, and stratification) to analyze the determinants of receiving a postpartum check while in the facility. Bivariate analysis (model 1) examined the association between each variable and the outcome, adjusted only for country-level random effects. All variables were retained regardless of significance in the subsequent models, except as noted. Model 2 adjusted for variables available in all surveys, excluding LOS at the facility (not available in eight countries), birthweight, and the two child-related characteristics (child sex and wantedness of child). Model 3 expanded on model 2 to include LOS. Finally, model 4 expanded on model 3 to include all variables. We also conducted a sensitivity analysis of model 4 by including the categorical child’s birthweight variable and reported on any substantial change in effect estimates. All regression models included a fixed effect for country, which adjusts for local context and for the fact that the outcome was based on three different question patterns. Additionally, we ran multilevel regression models of models 1–4 including random effects on the country level. The results were not substantively different, and the fixed-effect logistic regression results are shown.

Missing data were generally very low; child’s birthweight had the highest level of missingness (9.2% in the pooled sample). Early breastfeeding and ANC use had a low amount of missing data (3.4% and 2.4%, respectively), with all other determinants having less than 0.3% of observations missing. We used complete case analysis in the regression models.

This study did not register a prospective analysis plan; our analyses were guided from the onset by the two main study objectives. No data-driven changes to this strategy were made, with the exception of conducting a sensitivity analysis using random effects to understand whether the model provided substantively different results to the planned fixed effects model. This study is reported as per the Strengthening the Reporting of Observational Studies in Epidemiology (STROBE) guideline (S1 Checklist).

### Ethical approval

The DHSs receive government permission and follow ethical practices, including a written record of verbal informed consent and assurance of confidentiality. The Research Ethics Committee of the London School of Hygiene and Tropical Medicine approved our secondary-data analysis.

## Results

**Fig 2:**
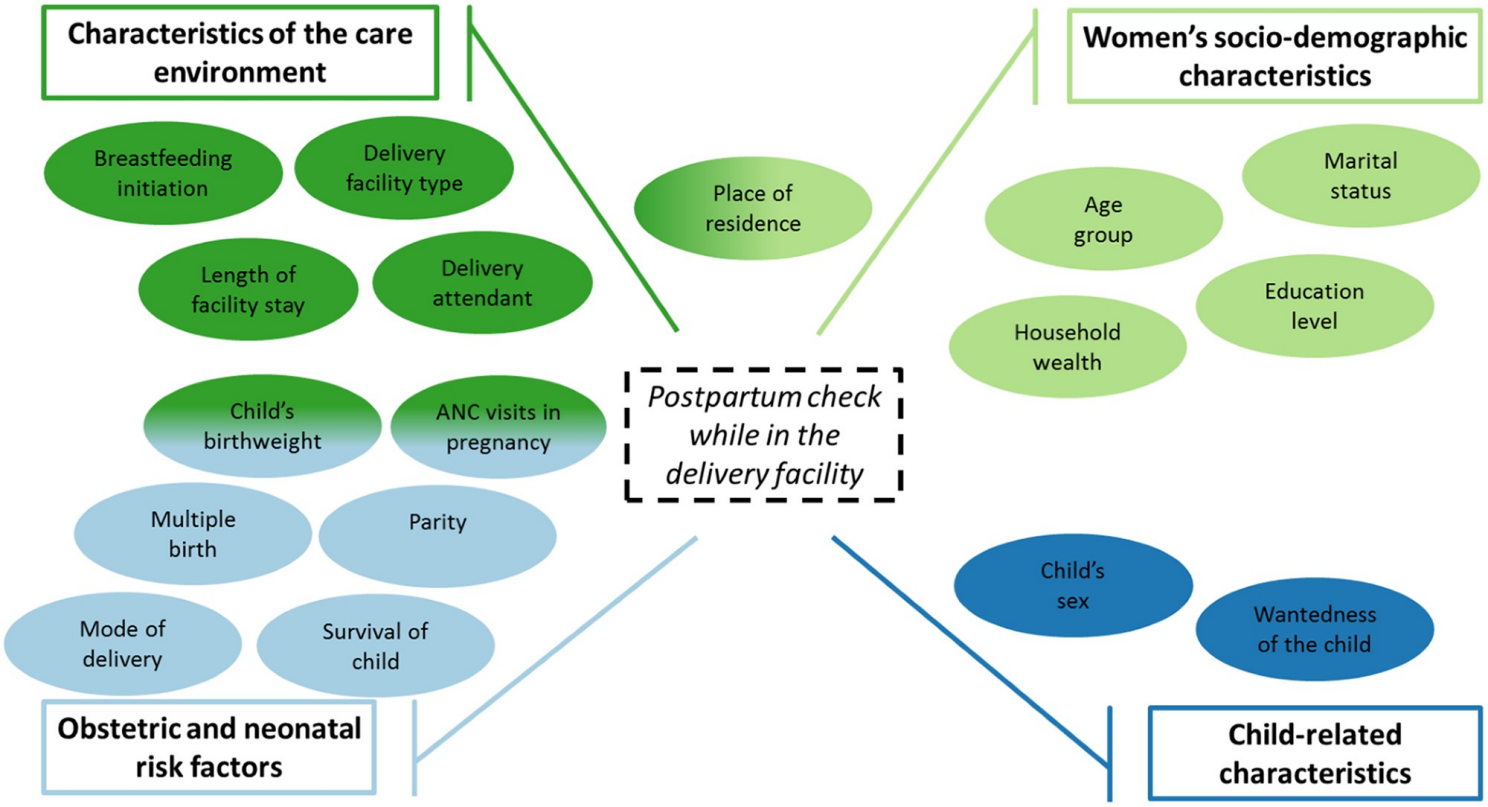
Percentage of women reporting receiving a postpartum check while in the facility following childbirth, by country and question pattern (A, light blue; B, dark blue; C, brown). ANC, antenatal care.

The analysis sample included a total of 137,218 women from 33 sub-Saharan African countries who had their most recent live birth in a health facility (question patterns A: 108,433, B: 9,896, and C: 18,889; S1 Table). The estimated percentage of women receiving a pre-discharge check across all countries was 66.6% (95% CI: 66.2–67.1). There was substantial variation between countries, with the median country percentage being 71.7% and a range from 26.6% in Eswatini (Swaziland at the time of survey) to 94.4% in Burkina Faso (Fig 2). The percentage of women with a pre-discharge check differed by the question pattern, with pattern C showing significantly lower percentages of being checked: A (67.8%, 95% CI: 67.3–68.3), B (67.4%, 95% CI: 66.3–68.5), and C (57.0%, 95% CI: 55.7–58.3), *p* < 0.001 (S1 Table). The ranges across the three question patterns were A: Burundi (43.8%) to Burkina Faso (94.4%); B: Swaziland (26.6%) to Madagascar (87.5%); and C: Chad (41.2%) to The Gambia (89.3%).

Based on the four conceptual categories of factors, we described the distribution of women in the pooled sample and the differences in the percentages of women receiving a pre-discharge check. All factors examined were significantly (*p* < 0.01) associated with reporting a pre-discharge check in the crude analysis except the survival status of the child and child’s sex (Table 2). The most extreme differences between categories were in factors describing the care environment or the obstetric/neonatal risk factors. Only 35.3% of women who reported a non-SBA as the highest cadre of childbirth attendant (a small category comprising 3.4% of the pooled sample) reported receiving a postpartum check while in the childbirth facility compared to 80.5% of women attended by a doctor/nonphysician clinician, an absolute percent point difference of over 45. Women who reported no ANC during pregnancy (2.8% of the pooled sample) were more than 20 percentage points less likely to report being checked compared to those receiving four or more ANC visits.

**Table 2:** Pooled sample, distribution of determinants and association between each determinant and receiving a postpartum check while in the childbirth facility (*n* = 137,218).

Conceptual Category	Factor (Number of Missing Observations)		% of Women by Category (Column %)	% of Women Checked by Category	*p*-Value (Chi-Squared)
Obstetric/ neonatal risk factors	Mode of delivery	Vaginal	91.7%	65.2%	<0.001
	(0)	Cesarean	8.3%	85.5%	
	Multiple birth	Singleton	97.7%	66.5%	0.004
	(0)	Twins, triplets	2.4%	71.0%	
	Survival of child	Died before/on day of discharge	1.0%	66.8%	0.058
	(0)	Survived	95.8%	66.7%	
		Died after discharge	3.3%	63.7%	
	Parity	First birth	23.9%	67.7%	<0.001
	(0)	2–3	35.7%	67.9%	
		4–6	28.6%	66.0%	
		≥7	11.8%	62.3%	
	Child weighed at birth	Yes	87.0%	67.8%	<0.001
	(12,806)	No	13.0%	54.1%	
	Child’s birthweight in grams	Extremely/very low (<1,499)	0.5%	67.4%	<0.001
	(among those weighed at birth)	Low (1,500–2,499)	8.2%	70.3%	
		Normal (2,500–4,000)	82.0%	67.9%	
		Macrosomia (>4,000)	9.3%	64.7%	
Woman’s sociodemographic characteristics	Age group at time of birth	<20	14.3%	63.4%	<0.001
	(0)	20–24	25.4%	65.0%	
		25–29	26.0%	67.8%	
		30–34	18.1%	69.0%	
		35–39	11.6%	67.9%	
		40–44	4.0%	67.4%	
		45–49	0.6%	64.3%	
	Marital status	Currently married	85.9%	67.0%	<0.001
	(2)	Never married	6.4%	67.9%	
		Formerly married	7.8%	61.2%	
	Highest level of education completed	No education	24.6%	67.6%	<0.001
	(18)	Primary	37.0%	59.7%	
		Secondary or higher	38.5%	72.6%	
	Household wealth quintile	Poorest	12.7%	58.7%	<0.001
	(0)	Poorer	16.4%	62.0%	
		Middle	19.1%	63.5%	
		Richer	23.3%	68.8%	
		Richest	28.6%	73.2%	
	Residence	Rural	57.0%	61.8%	<0.001
	(0)	Urban	43.0%	73.1%	
Child-related characteristics	Child sex	Male	51.0%	66.6%	0.781
	(0)	Female	49.0%	66.7%	
	Wantedness of child	Wanted	71.6%	68.7%	<0.001
	(90)	Mistimed	21.6%	61.8%	
		Unwanted	6.8%	60.1%	
Care environment characteristics	Delivery facility type	Public hospital	34.4%	74.5%	<0.001
	(0)	Public lower-level	46.9%	60.6%	
		Nonpublic (all levels)	18.7%	67.3%	
	Highest level of delivery attendant	Doctor/nonphysician clinician	17.0%	80.5%	<0.001
	(73)	Other SBA (nurse/midwife)	79.6%	65.0%	
		Non-SBA/no one	3.4%	35.3%	
	Breastfeeding initiation	Breastfed within 1 hour of delivery	33.4%	69.7%	<0.001
	(4,708)	Breastfed later/did not breastfeed	66.6%	65.1%	
			**Mean; Median (IQR)**	**Linear Regression b**	***p*-Value**
	ANC visits during pregnancy	continuous (0, 1, 2…,10+)	4.67; 4 (3,6)	0.029	<0.001
	(3,332)				
	LOS in facility	continuous (in days)	2.22; 1 (0,2)	0.010	<0.001
	(40,279; not available in eight countries)				

**Abbreviations**: ANC, antenatal care; LOS, length of stay; SBA, skilled birth attendant.

Women giving birth in public lower-level facilities (the most common category of childbirth location with 46.9% of the pooled sample) had the lowest level of pre-discharge check at 60.6% compared to those giving birth in nonpublic facilities (67.3%) and public hospitals (74.5%). Women who gave birth by a cesarean section (8.3% of the pooled sample) were more likely to have received a pre-discharge check (85.5%) compared to those with a vaginal birth (65.2%). Women who reported that their newborn was not weighed were much less likely to have been checked (54.1%) than women whose newborns were weighed (67.8%). We also found differences based on sociodemographic status, in which formerly married women (compared to those never and currently married), younger, poorer, rural, and high-parity women were less likely to have received a pre-discharge check. In the subsample of women from 25 countries that collected data on LOS in facility following childbirth, the data were right-skewed with a median of 1 day and mean of 2.22 days and showed a strong association between longer duration of stay and a higher likelihood of receiving a pre-discharge check.

We assessed the association of each factor with the outcome after adjusting for the effect of country (model 1 in Table 3). The largest effect sizes were seen for mode of delivery (cesarean odds ratio [OR] 3.54 compared to vaginal birth), richest wealth quintile (OR 1.89 compared to poorest), and delivery attendant being a non-SBA (OR 0.07 compared to doctor/nonphysician clinician). Every additional day spent in facility following childbirth was associated with 10% higher odds of receiving a pre-discharge check (*p* < 0.001). The findings of the three multivariable models were consistent. The most fully adjusted model (Table 3, model 4) shows a regression for the subsample of 25 countries where LOS was collected. Sensitivity analysis of model 4 for categories of birthweight did not produce an improved model fit (likelihood ratio test *p*-value 0.128).

**Table 3:** Crude and multivariable models of association between factors and receiving a postpartum check while in the childbirth facility, pooled sample.

	Model	1		2		3		4	
		Crude (adjusted for country only)		Adjusted for country + all variables except LOS, weighed at birth, and child sex and wantedness		Adjusted for country + all variables except weighed at birth and child sex and wantedness		Adjusted for country + all variables	
	Number of countries	33		33 (*n* = 128,933)		25 (*n* = 90,271)		25 (*n* = 81,373)	
Factor		OR (95% CI)	*p*-value*	OR (95% CI)	*p*-value**	OR (95% CI)	*p*-value**	OR (95% CI)	*p*-value**
Mode of delivery	Vaginal	ref		ref	<0.001	ref	<0.001	ref	<0.001
	Cesarean	3.54 (3.35–3.75)	<0.001	2.62 (2.46–2.80)		1.86 (1.70–2.02)		1.88 (1.72–2.05)	
Multiple birth	Singleton	ref		ref	0.001	ref	0.146	ref	0.197
	Twins, triplets	1.29 (1.19–1.42)	<0.001	1.18 (1.07–1.30)		1.09 (0.97–1.22)		1.08 (0.96–1.23)	
Survival of child	Died before/on day of discharge	1.02 (0.89–1.17)	0.788	0.99 (0.74–1.33)	0.082	0.87 (0.65–1.18)	0.046	0.95 (0.68–1.31)	0.288
	Survived	ref		ref		ref		ref	
	Died after discharge	0.87 (0.81–0.93)	<0.001	0.91 (0.84–0.99)		0.89 (0.80–0.98)		0.92 (0.82–1.02)	
Parity	First birth	ref		ref	0.167	ref	0.057	ref	0.512
	2–3	0.98 (0.95–1.01)	0.149	0.98 (0.94–1.02)		1.00 (0.95–1.04)		1.01 (0.96–1.07)	
	4–6	0.92 (0.89–0.95)	<0.001	0.95 (0.90–1.00)		0.95 (0.89–1.02)		0.98 (0.91–1.04)	
	≥7	0.87 (0.83–0.91)	<0.001	0.93 (0.86–1.00)		0.91 (0.83–0.99)		0.96 (0.87–1.05)	
Child weighed at birth	Yes	ref	<0.001					ref	<0.001
	No	0.39 (0.37–0.41)						0.54 (0.51–0.57)	
Woman’s age group at time of birth	<20	0.95 (0.90–0.98)	0.001	0.95 (0.91–1.00)	<0.001	0.95 (0.90–1.00)	<0.001	0.99 (0.93–1.04)	<0.001
	20–24	ref		ref		ref		ref	
	25–29	1.06 (1.02–1.10)	0.001	1.06 (1.02–1.10)		1.08 (1.03–1.13)		1.08 (1.02–1.13)	
	30–34	1.08 (1.04–1.12)	<0.001	1.10 (1.05–1.16)		1.12 (1.06–1.19)		1.12 (1.05–1.19)	
	35–39	1.10 (1.05–1.15)	<0.001	1.17 (1.10–1.24)		1.21 (1.13–1.30)		1.21 (1.12–1.31)	
	40–44	0.99 (0.93–1.06)	0.876	1.12 (1.02–1.21)		1.15 (1.04–1.26)		1.13 (1.02–1.25)	
	45–49	0.93 (0.78–1.10)	0.379	1.10 (0.92–1.32)		1.06 (0.85–1.33)		1.06 (0.83–1.34)	
Marital status	Currently married	ref		ref	0.105	ref	0.102	ref	0.081
	Never married	1.04 (1.00–1.10)	0.074	1.02 (0.97–1.08)		1.03 (0.96–1.10)		1.07 (1.00–1.14)	
	Formerly married	0.96 (0.92–1.00)	0.077	0.96 (0.91–1.00)		0.95 (0.89–1.00)		0.97 (0.91–1.03)	
Highest level of education completed	No education	ref		ref	<0.001	ref	<0.001	ref	<0.001
	Primary	1.12 (1.09–1.17)	<0.001	1.02 (0.99–1.07)		1.04 (0.99–1.09)		1.03 (0.97–1.08)	
	Secondary or higher	1.49 (1.44–1.55)	<0.001	1.12 (1.07–1.17)		1.12 (1.07–1.18)		1.11 (1.04–1.17)	
Household wealth quintile	Poorest	ref		ref	<0.001	ref	<0.001	ref	<0.001
	Poorer	1.10 (1.06–1.15)	<0.001	1.06 (1.02–1.11)		1.07 (1.01–1.13)		1.07 (1.01–1.13)	
	Middle	1.19 (1.14–1.24)	<0.001	1.08 (1.03–1.13)		1.06 (1.01–1.12)		1.06 (1.00–1.12)	
	Richer	1.42 (1.37–1.48)	<0.001	1.16 (1.11–1.22)		1.15 (1.09–1.22)		1.14 (1.07–1.20)	
	Richest	1.89 (1.81–1.97)	<0.001	1.31 (1.23–1.38)		1.36 (1.27–1.46)		1.32 (1.23–1.41)	
Residence	Rural	ref		ref	0.018	ref	<0.001	ref	0.001
	Urban	1.44 (1.41–1.48)	<0.001	1.04 (1.00–1.08)		1.10 (1.06–1.14)		1.08 (1.04–1.13)	
Child sex	Male	ref						ref	0.007
	Female	1.01 (0.99–1.03)	0.382					1.05 (1.01–1.08)	
Wantedness of child	Wanted	ref						ref	<0.001
	Mistimed	0.88 (0.85–0.90)	<0.001					0.87 (0.84–0.91)	
	Unwanted	0.85 (0.81–0.89)	<0.001					0.79 (0.74–0.85)	
Delivery facility type	Public hospital	ref		ref	<0.001	ref	<0.001	ref	0.002
	Public lower-level	0.63 (0.61–0.64)	<0.001	0.85 (0.82–0.88)		0.93 (0.89–0.96)		0.94 (0.90–0.98)	
	Nonpublic (all levels)	0.97 (0.93–1.00)	0.119	0.99 (0.95–1.04)		0.99 (0.94–1.04)		1.02 (0.97–1.08)	
Highest level of delivery attendant	Doctor/nonphysician clinician	ref		ref	<0.001	ref	<0.001	ref	<0.001
	Other SBA (nurse/midwife)	0.47 (0.45–0.49)	<0.001	0.70 (0.67–0.73)		0.73 (0.69–0.78)		0.74 (0.69–0.78)	
	Non-SBA	0.07 (0.07–0.08)	<0.001	0.12 (0.11–0.13)		0.13 (0.12–0.14)		0.14 (0.12–0.15)	
Breastfeeding initiation	Breastfed within 1 hour of delivery	ref		ref	0.012	ref	0.001	ref	0.006
	Breastfed later/did not breastfeed	1.07 (1.04–1.10)	0.002	0.96 (0.93–0.99)		0.94 (0.91–0.98)		0.95 (0.91–0.98)	
ANC visits during pregnancy	(continuous: 0, 1, 2, …, 10+, odds associated with one visit increase)	1.09 (1.09–1.11)	<0.001	1.06 (1.05–1.06)	<0.001	1.06 (1.04–1.06)	<0.001	1.04 (1.03–1.05)	<0.001
LOS in facility	(continuous, odds associated with 1-day increase)	1.10 (1.09–1.10)	<0.001			1.06 (1.05–1.07)	<0.001	1.06 (1.05–1.07)	<0.001

*p-value of Wald test.

**p-value of likelihood ratio test.

**Abbreviations**: ANC, antenatal care; CI, confidence interval; LOS, length of stay; OR, odds ratio; ref, reference; SBA, skilled birth attendant.

Model 4 shows that factors from all four conceptual categories were significant determinants of receiving a pre-discharge check. In terms of obstetric/neonatal risk factors, women with a cesarean section had 88% higher odds of being checked before discharge. Parity and infant survival were not associated with pre-discharge check. Mothers of newborns who were not weighed were 46% less likely to have received a check before discharge. Among the sociodemographic characteristics, age 25 to 44 years was associated with 8%–21% increase in the odds of being checked compared to the 20- to 24-year–old reference group. Women in the wealthier four quintiles and those with secondary and higher education were more likely to receive a pre-discharge check compared to the poorest fifth and those with less education. Urban residents were 8% more likely to be checked compared to rural dwellers. Women with mistimed or unwanted pregnancies were significantly less likely to have received a pre-discharge check compared to women with wanted pregnancies. In the conceptual category of care environment, all five assessed determinants were significantly associated with receiving a pre-discharge check. Women giving birth in lower-level public facilities (compared to those in public hospitals), those attended by a nurse/midwife or a non-SBA (compared to doctor/nonphysician clinician), and women who did not initiate breastfeeding within an hour of birth (compared to those who did) had lower adjusted odds of receiving a pre-discharge check. Every additional ANC visit during pregnancy increased the odds of a pre-discharge check by 4% and additional day in the childbirth facility by 6%.

## Discussion

This is the first study, to our knowledge, to use nationally representative data from multiple sub-Saharan African countries to examine within-facility postpartum care to women. Based on recent DHS data from 33 countries, we found that the percentage of women reporting receipt of a pre-discharge check by a health professional following childbirth in a health facility ranged widely across countries and was far from universal. In only four countries—Burkina Faso, Ivory Coast, Ghana, and Sierra Leone—did more than 90% of women report receiving this check. In 18 of the 33 countries, fewer than three-quarters of women received this essential element of care. A previous analysis of levels, timing, and providers of postnatal care by place of birth in eight countries in SSA showed similarly suboptimal and widely ranging levels of coverage (the percentage of women who gave birth in facilities who received postpartum care within 41 days of birth ranged from 41% in Uganda to 92% in Ghana) [21]. However, this study did not assess whether a postnatal check occurred while women were still in health facilities, nor did it explore the determinants of receiving such care.

We found that more educated, wealthier women and those who received more ANC visits were more likely to receive a pre-discharge check. This may be related to greater awareness of potential complications but could also suggest that these women have greater agency to ask to be checked by a health provider or perhaps better awareness of what constitutes a check and ability to recall the event [22,23]. Our study uniquely used data from more countries than other studies on the subject, to our knowledge, and the resulting larger pooled analysis sample enabled the examination of rare factors such as child survival and multiple births. Surprisingly, we found no difference in reporting receipt of the pre-discharge check among mothers of multiples compared to singletons or among mothers of children who survived and those who died before or after discharge. Neither was the coverage with pre-discharge check universal among women who had a cesarean section. This suggests that maternal and neonatal risk factors might not prompt providers to provide comprehensive care to women.

Discharge procedures are likely to vary across facilities. Urban residents were more likely to receive a pre-discharge check, which may indicate more formal discharge systems because the greater volume of births at urban facilities results in the need for rapid bed turnover. Additionally, in some facilities, women may discharge themselves before receiving a check, particularly if the additional clinical examination or time spent in facility incurs additional out-of-pocket cost [24]. Women giving birth with a doctor/nonphysician clinician were more likely to receive a pre-discharge check, though this might signify more complicated births. It is possible that women did not recognize the pre-discharge check as separate from the care during labor/birth or from check on the newborn, especially women whose births were attended by nurses or midwives. The small percentage of women who reported being assisted by a non-SBA during childbirth had 86% lower odds of pre-discharge check compared to those attended by a doctor/nonphysician clinician. While women’s ability to accurately distinguish and report the exact cadre of birth attendant is imperfect [25–29], this factor may capture larger issues of insufficient staffing, poor training, and low adherence to guidelines in certain facilities. Our findings show that ANC, delivery with an SBA, and longer LOS are associated with a postpartum check before discharge from a health facility, thus highlighting the critical need for continuous quality of care along the continuum of maternal and child health services [30].

As documented previously[18], the very short median LOS in facilities after childbirth, in the absence of home visits and follow-up at the community level, likely leaves some complications such as postcaesarean surgical site infection undiagnosed and at risk of not receiving proper care and follow-up. We note the higher odds of reporting a pre-discharge check among women with longer lengths of stay in facilities, which is potentially due to such cases being more complicated and requiring more intensive care and attention or because the need for a check before discharge is driving women to remain longer.

The heterogeneity in postpartum coverage indicators shows important differences in practices across settings, even for neighboring countries. These differences might be explained by numerous and multifaceted factors, including the heterogeneity of countries’ perinatal policies and systems; uneven levels of facility-based delivery; differences in healthcare providers assisting childbirth; heterogeneous quality of care; and additional determinants pertaining to contexts, values, and experiences of postpartum care. Our study adjusts for some of these factors, but the cross-country variability remains highly significant. Further research is needed to understand this heterogeneity and assess the underlying causes of different practices across countries in SSA.

### Limitations

This study was limited by the accuracy of the respondent’s recall of the provider, timing, and receipt of postpartum checks for births up to 5 years prior to interview; we only included the respondent’s most recent birth to reduce the extent of recall error. A study of women’s recall of key postnatal events showed acceptable levels in two sub-Saharan African settings [31]. The outcome of interest was measured using slightly different questions across the 33 included countries. In the majority of included surveys, women were asked questions according to pattern A, which does not define what constitutes a “check,” compared to pattern B, which specifically asks about a “physical examination” before discharge. Women in different countries might have understood the term “check” variably. LOS was not available in all countries included in this analysis, although it was available in countries following question pattern C, where it was needed to determine whether the postpartum check took place before or after discharge from facility. While efforts were made to adjust for this variation in ascertainment of outcome in multivariate analysis by including country-level fixed effects, women’s interpretations of the differing questions could have biased our results.

DHSs do not consistently ask women across all country surveys how long after childbirth or how long before discharge they were checked, how many times, or what actions constituted this check [32]. We also do not know whether appropriate action was taken on the basis of the examination or how postpartum care was continued in the community after discharge from facility. Importantly, we did not have information on the women’s health status, complications surrounding the delivery, or the newborn’s gestational age and were unable to adjust for these factors in our models. While mode of delivery, multiple birth, child survival status, and LOS capture some dimensions of the need for more intensive monitoring, the final estimates might still reflect residual confounding.

A further limitation is the availability of data, which were collected between 2006 and 2015. Some of the cross-country variability in the levels of the main outcome might be attributable to this. While the majority of surveys included were conducted recently, for three countries—Madagascar (2009), Sao Tome and Principe (2008–2009), and Swaziland (2006)—the most recent DHS survey was conducted prior to 2010. As such, any recent improvements or changes in provision of postpartum care are not captured in the data. There was low extent of missingness except for birthweight, which was missing for approximately 9% of records in the pooled sample. Despite these limitations, this study provides an in-depth examination of a critical and understudied component of maternal healthcare and is an important contribution to the literature on quality of childbirth care provided in facilities in LMICs.

The low coverage of pre-discharge checks in facilities in many of the included sub-Saharan African countries may reflect the lack of importance given to postpartum care in the global discourse on essential interventions and quality improvement in maternal health [33]. While tracer indicators appeared in the Millennium Development Goals and again in the Sustainable Development Goals for coverage of 4+ ANC visits and skilled care at birth, no single corresponding global indicator currently exists for maternal postpartum care [34,35]. WHO postpartum care guidelines exist [8]; however, the lack of a global focus on the mother’s care in the postpartum period, including fewer evidence-based algorithms, guidelines, and the absence of routine monitoring indicators, may lead to fragmented interpretations of what is considered “essential” maternal care at the country level. Our study shows the critical gap between the recommended postnatal checks within the first 24 hours and before discharge [8,9], thus highlighting the unmet needs of these women who are too often “left behind” by health systems in LMICs. Further action is needed to enhance the integration and quality of postnatal care services for mothers and neonates, and the critical unmet need in postnatal care should be addressed in policy, practice, and research.

Recent studies in high-income countries posited that a greater focus on the newborn has resulted in less attention to the postpartum needs of women. For example, a survey of postpartum nurses in the United States found low knowledge of postpartum maternal complications, and many did not comprehensively counsel patients prior to discharge [36]. In LMICs, the lack of a pre-discharge check may partly be a result of inadequate staffing, particularly in lower-level facilities, where odds of receiving the check were lower because providers are busy assisting other women in labor or with complications [15,37]. Already overburdened or burned-out staff [38] may see postpartum care and pre-discharge checks as a less important priority compared to providing antenatal and intrapartum services [11].

Our results also suggest the assumption made by other studies and estimates of postnatal care coverage, that all women giving birth in health facilities receive early postpartum care, is not borne out by the data. In light of substantial increases in institutional deliveries, greater attention is needed to ensure continuous, high-quality, and equitable care in facilities [39,40]. The low levels of pre-discharge checks in many countries suggest insufficient attention to this crucial component in the continuum of pregnancy, intrapartum, and postpartum care. Additionally, where facility-based childbirth rates are low, postnatal care in the community for births outside of facilities is crucial but likely to be even worse [21].

### Conclusion

Addressing disparities in access to both facility-based childbirth and good-quality postpartum care in SSA is critical to confronting stalling declines in maternal mortality and reducing maternal complications that leave women with lifelong negative health sequelae. Our findings highlight the need for greater consideration to postpartum care, particularly postpartum care after births in health facilities, in research, policy, and practice in SSA. Additional consideration should be provided to effective coverage of postpartum care, including functions, content, and quality of care. Pre-discharge checks are a key opportunity to advocate for greater use of services across the full 6-week postpartum period, with a view to enhancing continuity of care and the integration of maternal and newborn services. Similar to how recent WHO guidelines recommend ANC and intrapartum services to facilitate a positive pregnancy experience, recommendations for more structured and person-centered postnatal care contacts could complete the pregnancy cycle and improve both the woman’s experience and health outcomes. These considerations are now being addressed by the ongoing update to WHO guidelines on postnatal care. Global and country-level policymakers should consider guidelines and strategies that ensure that provision of quality postpartum care to women is prioritized along the continuum of maternal, newborn, and child health.

## Supporting information

S1 Table List of countries, survey year, question pattern, distribution of location of childbirth, and estimate of percentage of women receiving a postpartum check before discharge among women whose most recent live birth was in a health facility. (DOCX)

S1 Checklist STROBE Checklist. STROBE, Strengthening the Reporting of Observational Studies in Epidemiology.
(DOC)

## Abbreviations

ANC: antenatal care
aOR: adjusted odds ratio
CI: confidence interval
DHS: Demographic and Health Survey
LMICs: low- and middle-income countries
LOS: length of stay
OR: odds ratio
SBA: skilled birth attendant
SSA: sub-Saharan Africa
STROBE: Strengthening the Reporting of Observational Studies in Epidemiology
WHO: World Health Organization
