# Supplementary material for: Provision of postpartum care to women giving birth in health facilities in sub-Saharan Africa: A cross-sectional study using Demographic and Health Survey data from 33 countries
Source: PLoS Med. 2019 Oct 23;16(10):e1002943. doi: 10.1371/journal.pmed.1002943 (PMC6808422; doi:10.1371/journal.pmed.1002943)
Supplement: S1 Table — (DOCX) [file pmed.1002943.s001.docx]

S1 Table. List of countries, survey year, question pattern, distribution of location of childbirth, and estimate of percentage of women receiving a postpartum check before discharge, among women whose most recent live birth was in a health facility
